# Multifaceted Applications of Zerumbone-Loaded Metal–Organic Framework-5: Anticancer, Antibacterial, Antifungal, DNA-Binding, and Free Radical Scavenging Potentials

**DOI:** 10.3390/molecules30142936

**Published:** 2025-07-11

**Authors:** Sumeyya Deniz Aybek, Mucahit Secme, Hasan Ilhan, Leyla Acik, Suheyla Pinar Celik, Gonca Gulbay

**Affiliations:** 1 Department of Medical Biology, Faculty of Medicine, Ordu University, Ordu 52200, Türkiye; mucahitsecme@odu.edu.tr (M.S.); goncagulbay@odu.edu.tr (G.G.); 2 Department of Biotechnology, Institute of Biotechnology, Ankara University, Ankara 06110, Türkiye; ilhanhasan81@gmail.com; 3 Department of Biology, Faculty of Science, Gazi University, Ankara 06500, Türkiye; leylaacik@gazi.edu.tr (L.A.); pinarsuheylacelik@hotmail.com (S.P.C.)

**Keywords:** breast cancer, drug delivery systems, metal–organic framework, nanoparticles, zerumbone

## Abstract

In the present research, metal–organic framework-5 (MOF-5) was synthesized and loaded with zerumbone (ZER@MOF-5), followed by the evaluation of its anticancer, antibacterial, antifungal, DNA-binding, and free radical scavenging potentials. The synthesized nanoparticles were characterized using X-ray diffraction, ultraviolet–visible spectroscopy, Fourier-transform infrared spectroscopy, energy-dispersive X-ray spectroscopy, and scanning electron microscopy. The *in vitro* anticancer activity of ZER@MOF-5 was studied in a human breast cancer cell line (MCF-7) using the CCK-8 assay. The interaction of ZER@MOF-5 with pBR322 plasmid DNA was assessed by gel electrophoresis. The antimicrobial effect of ZER@MOF-5 was examined in gram-positive and gram-negative bacterial strains and yeast strains using the microdilution method. The free radical scavenging activity was assessed using the DPPH assay. Cytotoxicity assay revealed a notable enhancement in the anticancer activity of zerumbone upon its encapsulation into MOF-5. The IC_50_ value for ZER@MOF-5 was found to be 57.33 µg/mL, which was lower than that of free zerumbone (IC_50_: 89.58 µg/mL). The results of the DNA-binding experiment indicate that ZER@MOF-5 can bind to target DNA and cause a conformational change in DNA. The results of the antibacterial activity experiment showed that the antibacterial ability of ZER@MOF-5 was limited compared to free zerumbone. The results of the DPPH assay demonstrated that the antioxidant activity of free zerumbone was higher than that of ZER@MOF-5. MOFs encapsulate compounds within their porous crystalline structure, which leads to prolonged circulation time compared to single ligands. Although the unique structure of MOFs may limit their antibacterial and antioxidant activity in the short term, it may increase therapeutic efficacy in the long term. However, to fully understand the long-term antibacterial and antioxidant effects of the ZER@MOF-5, further comprehensive *in vitro* and *in vivo* experiments are necessary. This finding indicates that the MOF-5 could potentially be an impressive carrier for the oral administration of zerumbone.

## 1. Introduction

Cancer is the leading cause of morbidity and mortality, resulting in a substantial burden of disease. The Global Cancer Statistics for 2022 reported nearly 20 million new cancer cases in 2022, alongside 9.7 million deaths from it [1]. Patients often suffer from poor efficiency and serious side effects such as severe toxicity to normal tissues, drug resistance, and severe metastasis, followed by traditional anticancer therapies [2]. Bacterial infections are one of the most serious complications associated with cancer therapy. Patients with cancer face a high risk of these infections due to factors such as radiotherapy and chemotherapy-related neutropenia, surgical complications, and immunosuppressive therapies. Despite the ongoing decline in cancer mortality rates, bacterial infections continue to be a major cause of death in cancer patients [3]. In this context, intense efforts are being made within the health and pharmaceutical sector to find a single, more effective drug capable of treating both cancer and bacterial infections that occur during cancer treatment.

**Figure 1:**
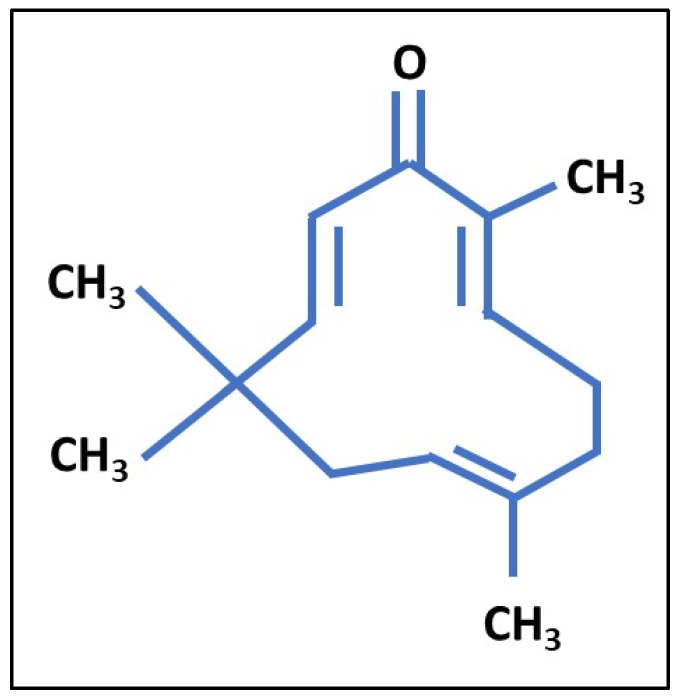
Chemical structure of zerumbone.

Zerumbone is a multifunctional natural compound that is extracted from the rhizomes of *Zingiber zerumbet*. It contains three double bonds (two conjugated and one isolated): α, β-unsaturated carbonyl group, and a double-conjugated carbonyl group in the 11-membered ring structure. The molecular formula for zerumbone is C_15_H_22_O (Figure 1). This natural compound possesses biological properties, including anticancer, antimicrobial, anti-inflammatory, and antioxidant activity. The preclinical applications of zerumbone are limited by low oral bioavailability, which is associated with low permeability and poor aqueous solubility [4,5]. As a solution to this, nanoparticles have been developed to achieve more efficient delivery of zerumbone.

Nanotechnology-based drug delivery systems (DDS) offer distinct benefits for the delivery of therapeutic agents, allowing for cargo protection, co-delivery, the targeting of specific cells and tissues, and a reduction in their lateral effects [6]. Indeed, the convergence of nanotechnology and medicine holds great promise for addressing the complex challenges associated with cancer treatment and bacterial infections that often arise during therapy. These technologies, which include self-assembled materials and nano/microparticles, have been extensively studied in the contexts of cancer and bacterial infections [7,8,9,10].

Nanostructured lipid carriers (NLCs) are a promising strategy for encapsulation of lipophilic drugs like zerumbone; these nanocarriers offer extensive drug loading, stability, and better release profile [11]. Rahman et al. found that *in vitro* drug release of zerumbone from the ZER-NLC was slower than pure zerumbone dispersion [12]. Nanosuspension is another strategy to improve the solubility of zerumbone. Md et al. formulated a nanosuspension of zerumbone to improve its dissolution profile [4]. The cyclodextrin inclusion complex has been widely investigated in novel drug formulations because of its ability to improve the water solubility of hydrophobic drugs. Eid et al. [13] reported that the water solubility of zerumbone increased more than 30-fold by zerumbone encapsulated with hydroxypropyl-*β*-cyclodextrin (ZER-HP*β*CD). Another study demonstrated that the inclusion complex formation of zerumbone and different cyclodextrin derivatives enhanced stability, solubility, and bioavailability [14]. Collectively, these studies suggest that drug nanoparticles are useful to improve the bioavailability of zerumbone.

Metal–organic framework-5 (MOF-5) is one member of the broader class of metal–organic frameworks (MOFs), which have attracted great attention due to their large specific surface area and high porosity. MOF-5, also known as IRMOF-1, is a three-dimensional cubic porous framework, which consists of [Zn_4_O]^6+^ clusters connected by 1,4-benzenedicarboxylate (BDC^2−^) ligands [15]. Recently, MOF-5 holds great promise for clinical applications of drugs owing to its potential biodegradability and multifunctionality [16]. Compared to conventional nanocarriers, MOFs offer distinct advantages. Drug molecules can be easily loaded on the surface of MOFs via electrostatic interaction, surface coordination, and π–π stacking interaction, and then these molecules can diffuse into the MOF pores and be effectively loaded via interacting with metal clusters or organic linkers [17]. MOFs exhibit remarkably high drug-loading capacities and tunable drug release properties compared to liposome-based platforms. Furthermore, the modular nature of MOFs allows functionalization with organic ligands or various metal centers, thereby enabling the construction of multifunctional drug delivery platforms [18]. In the context of zerumbone delivery, MOF-5 was selected due to its well-characterized structure, high surface area, and proven capacity for drug encapsulation and controlled release.

To our knowledge, there are no studies investigating the anticancer, antibacterial, antifungal, and free radical scavenging potentials of metal–organic framework-5 to load zerumbone (ZER@MOF-5). The purpose of the present study is to fabricate ZER@MOF-5 and evaluate its anticancer, antibacterial, antifungal, DNA-binding, and free radical scavenging activities. For this purpose, we successfully fabricated MOF-5 and loaded it with zerumbone. The synthesized nanoparticles were characterized using X-ray diffraction (XRD), ultraviolet–visible (UV-Vis) spectroscopy, Fourier-transform infrared (FTIR) spectroscopy, energy-dispersive X-ray (EDX) spectroscopy, and scanning electron microscopy (SEM). The antimicrobial effect of newly synthesized ZER@MOF-5 was tested against various bacteria and yeast strains using the microdilution method. The DNA-binding activity of ZER@MOF-5 nanoparticles was examined via gel electrophoresis. The free radical scavenging activity was assessed using the 2,2-diphenyl-1-picrylhydrazyl (DPPH) assay. The *in vitro* anticancer activity of ZER@MOF-5 was studied in a human breast cancer cell line (MCF-7) using the CCK-8 assay.

## 2. Results

### 2.1. Characterization of the MOF-5 and ZER@MOF-5

**Figure 2:**
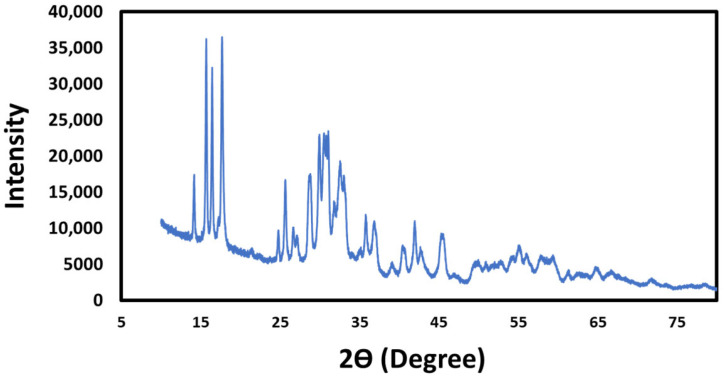
X-ray Diffraction (XRD) patterns for the synthesized MOF-5.

As shown in Figure 2, the XRD pattern of the synthesized MOF-5 exhibits a series of sharp and well-defined diffraction peaks spanning the 2θ range of approximately 6° to 30°, indicating a high degree of crystallinity and a well-ordered cubic framework. The most intense peak is observed at ~6.8°, which corresponds to the (200) reflection and is characteristic of MOF-5. Additional peaks appear at ~9.6°, 13.7°, 15.4°, and several higher-order reflections around 17.0°, 18.4°, 20.8°, 24.4°, and 26.7°, among others. These peaks are consistent with the reported XRD patterns of cubic MOF-5 (Fm-3m space group) in the literature and collectively confirm the formation of a phase-pure and highly crystalline material. The absence of any extra peaks in the 2θ range of 30–40°, such as those typically associated with ZnO (e.g., at 31.5°, 34.6°, and 36.1°), further supports the phase purity of the product. These findings are in agreement with the Powder X-ray diffractometer (P-XRD) profiles reported by Chen et al. [19], who have established the peak intensity ratios and positions as reliable indicators of framework integrity and interpenetration.

**Figure 3:**
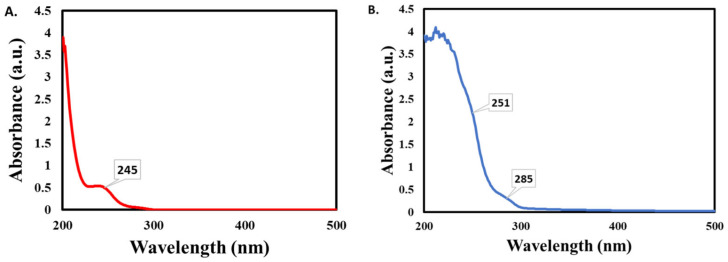
(**A**) UV–vis spectrum of MOF-5 and (**B**) UV–vis spectrum of ZER@MOF-5.

UV–vis spectroscopy analysis was employed to investigate the optical absorption properties of both pure MOF-5 and ZER@MOF-5. As shown in Figure 3A, pure MOF-5 exhibits a distinct absorption peak centered at approximately 245 nm, which is characteristic of π–π* transitions associated with its organic linker, terephthalic acid [20]. Upon successful loading of zerumbone into the MOF-5 framework, notable spectral changes were observed. The ZER@MOF-5 spectrum displayed a red-shifted shoulder around 251 nm, suggesting interactions between Zerumbone and the MOF-5 matrix (Figure 3B). More prominently, a new absorption peak emerged at 285 nm, which can be attributed to the π–π* or n–π* transitions originating from the conjugated carbonyl and α, β-unsaturated systems of zerumbone. This spectral shift and the appearance of new features provide strong evidence for the successful encapsulation of zerumbone within the MOF-5 structure and suggest possible electronic interactions between the guest molecule and the host framework.

**Figure 4:**
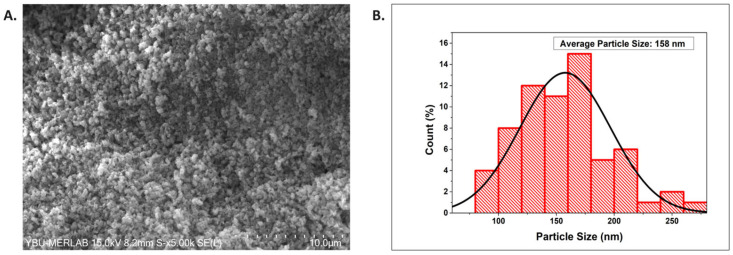
(**A**) Scanning Electron Microscopy (SEM) images of the MOF-5 and (**B**) particle size distribution histogram of MOF-5 obtained from SEM analysis.

**Figure 5:**
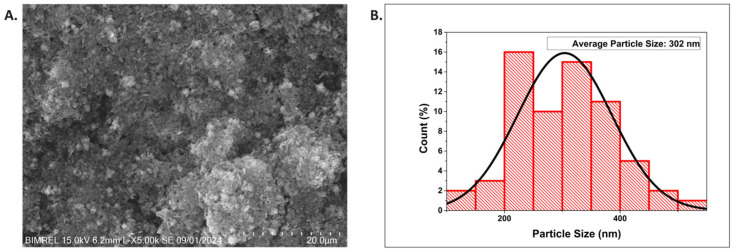
(**A**) Scanning Electron Microscopy (SEM) image of ZER@MOF-5 and (**B**) particle size distribution histogram of ZER@MOF-5 obtained from SEM analysis.

The crystalline structure of pure MOF-5 was visualized by SEM analysis, which was consistent with the result obtained from the XRD pattern [21]. The average particle size of MOF-5 was calculated to be approximately 158 nm, as shown in Figure 4. The SEM image of ZER@MOF-5 revealed slightly aggregated spherical and irregular clusters, maintaining the overall porous morphology. The surface appeared denser and more textured, which may be attributed to the presence of encapsulated zerumbone. Larger clusters observed in the image likely indicate areas of higher zerumbone concentration, confirming effective loading. The average particle size of ZER@MOF-5 was calculated to be approximately 302 nm. Importantly, the structural integrity of the MOF-5 framework remained intact after loading, confirming that the encapsulation process did not significantly disrupt its morphology (Figure 5).

### 2.2. Energy-Dispersive X-Ray Spectroscopy

**Figure 6:**
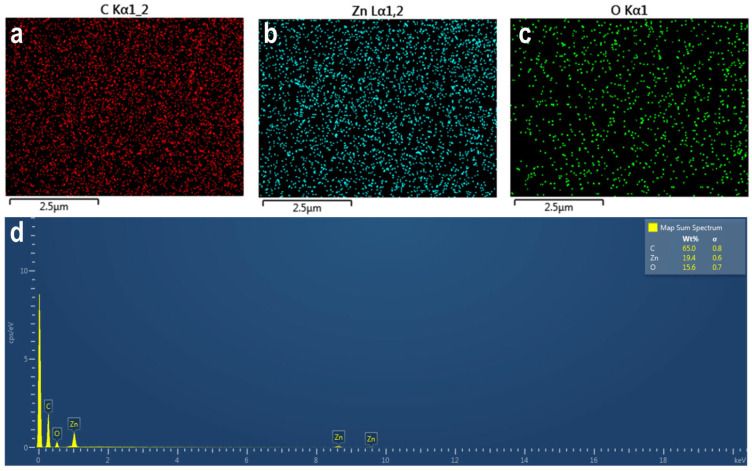
Scanning electron microscopy (SEM)-Energy-dispersive X-ray spectroscopy (EDX) mapping results of the ZER@MOF-5. (**a**) Carbon (C) map, (**b**) zinc (Zn) map, (**c**) oxygen (O) map, and (**d**) the EDX spectrum obtained from the ZER@MOF-5.

Elemental mapping was conducted to assess the spatial distribution of carbon (C), zinc (Zn), and oxygen (O) within the ZER@MOF-5 (Figure 6a–c). The carbon map showed a uniform distribution of carbon throughout the sample, which suggests that zerumbone is well-dispersed within the MOF-5 structure. This is crucial for the uniformity and effectiveness of the material’s intended applications. Similarly, the zinc map displayed a homogeneous spread of zinc across the area, consistent with the expected uniformity of the MOF-5 framework. The oxygen map also indicated an even distribution of oxygen, supporting the integration of zerumbone within the MOF-5, as both the organic component and the MOF’s framework contribute to the overall oxygen content. The uniform distribution of carbon, zinc, and oxygen observed in the elemental maps confirms the successful loading of zerumbone into the MOF-5 structure.

The elemental composition and distribution of the ZER@MOF-5 are shown in Figure 6d. The EDX spectrum of the ZER@MOF-5 revealed the presence of carbon, zinc, and oxygen as the primary elements in the sample. The quantitative analysis showed that carbon was the predominant element, with a weight percentage of 64.99% and an atomic percentage of 80.95%, indicating the significant contribution of the organic components from zerumbone and the MOF-5 structure. Zinc, a key element of the MOF-5 framework, was present at a weight percentage of 19.38% and an atomic percentage of 4.44%. Oxygen was also detected, with a weight percentage of 15.63% and an atomic percentage of 14.62%, corresponding to both the MOF structure and the organic functional groups from zerumbone. The uniform distribution of carbon, zinc, and oxygen observed in the elemental maps and EDX spectrum confirms the successful loading of zerumbone into the MOF-5 structure.

### 2.3. Analysis of FTIR Spectroscopy

**Figure 7:**
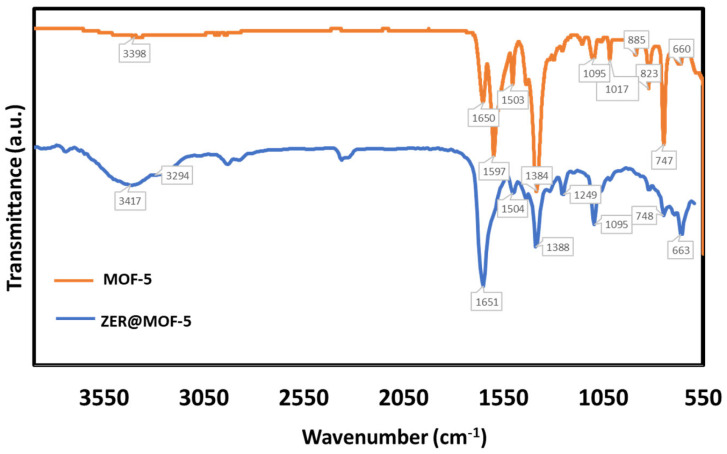
FTIR spectrum of the MOF-5 and ZER@MOF-5.

The FTIR spectra of ZER@MOF-5 and pure MOF-5 exhibit significant differences, providing evidence of successful zerumbone loading into the MOF-5 framework (Figure 7). In the pure MOF-5 spectroscopy, characteristic peaks are observed at 3398 cm^−1^, indicating low-intensity O-H stretching vibrations, likely due to residual water molecules or hydroxyl groups within the MOF pores. A strong peak at 1650 cm^−1^ is attributed to the C=O stretching of the terephthalate linker, an essential component of the MOF-5 structure. Additionally, peaks at 1597 and 1384 cm^−1^ are associated with aromatic C=C stretching vibrations from the aromatic rings within the terephthalate linker. Peaks at 1095, 1017, 885, 823, 747, and 660 cm^−1^ correspond to Zn–O stretching and bending modes, which are characteristic of the metal–organic framework and confirm the presence of the inorganic ZnO framework in MOF-5. The FTIR spectrum of pure MOF-5 is in good agreement with the literature [22].

In contrast, the ZER@MOF-5 spectrum shows several shifts and additional peaks that confirm the successful loading of zerumbone. The O-H stretching region shifts to 3417 cm^−1^ and 3294 cm^−1^, suggesting possible hydrogen bonding interactions between zerumbone molecules and the MOF structure, which may arise from hydroxyl groups on zerumbone interacting with MOF-5. The strong C=O stretching peak shifts slightly to 1651 cm^−1^, indicating interactions between the carbonyl groups of zerumbone and the MOF framework. The peak appears at 1504 cm^−1^, which can be attributed to C=C stretching or in-plane bending associated with the zerumbone structure, suggesting that the aromatic framework of zerumbone is present and interacting within MOF-5. Additional peaks at 1388 and 1249 cm^−1^ are likely due to C-H bending and other functional groups in zerumbone, further confirming its presence within the MOF structure. Peaks at 1095, 748, and 663 cm^−1^ are still observed, indicating that the Zn–O framework of MOF-5 remains intact despite the encapsulation of zerumbone.

In conclusion, the comparative FTIR analysis shows distinct spectral shifts and new peaks in the ZER@MOF-5 spectrum, indicating successful loading. The shifts in O-H and C=O regions, along with additional peaks corresponding to functional groups of zerumbone, confirm interactions between zerumbone and the MOF-5 framework. These changes verify the effective encapsulation of zerumbone within the MOF-5 structure.

### 2.4. Antimicrobial Activity

**Figure 8:**
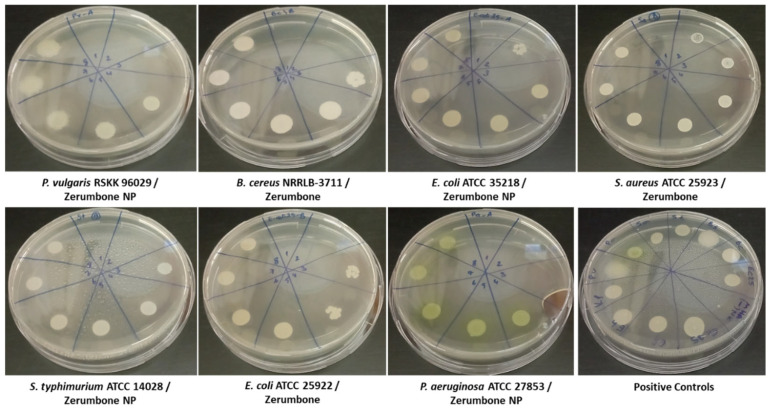
Antimicrobial activities of zerumbone and ZER@MOF-5 (Zerumbone NP).

As shown in Table 1, the results of MIC indicated that the zerumbone exhibited high activity against the tested bacteria. Although its activity against *B. subtilis* was lower than against other strains, it was still more potent than that of ampicillin. Antifungal activities of the zerumbone and ZER@MOF-5 were presented in Table 1. The zerumbone showed the highest activity against the *C. albicans*, *C. krusei*, and *C. tropicalis* compared to positive controls, while the ZER@MOF-5 showed lower activity against the *C. albicans*, *C. krusei*, and *C. tropicalis* compared to positive controls. The results of MBC and MFC of the zerumbone and ZER@MOF-5 are presented in Table 2 and Figure 8. The results showed that the zerumbone was the most efficient in inhibiting the growth of all evaluated bacteria. Although its activity against *B. subtilis* was lower than against other strains, it was still more potent than that of ampicillin. It was also the most efficient in inhibiting the growth of all evaluated fungal strains compared to the ZER@MOF-5 and positive controls.

### 2.5. Interactions of pBR322 Plasmid DNA with the ZER@MOF-5

**Figure 9:**
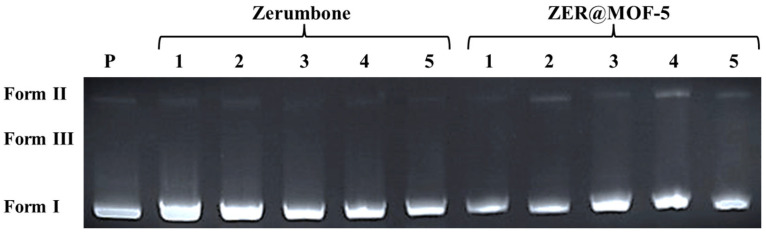
Electrophoretograms of the interaction between pBR322 plasmid DNA and decreasing concentrations of the zerumbone and ZER@MOF-5. Lane P applies to untreated pBR322 plasmid DNA (control). Concentrations (in mg/mL) are as follows: lanes 1–5 apply to plasmid DNA interacted with decreasing concentrations of the Zerumbone and ZER@MOF-5: lane 1: 10; lane 2: 5; lane 3: 2.5; lane 4: 1.25; lane 5: 0.625. The roman numerals I, II, and III indicate form I (supercoiled), form II (open circular), and form III (linear) plasmids, respectively.

The interaction of the zerumbone and ZER@MOF-5 with pBR322 plasmid DNA was observed via gel electrophoresis. The binding of a compound to DNA can alter the conformation of the DNA and lead to a noticeable change in electrophoretic mobility. If there is a strong interaction between DNA and compounds, this can cause DNA cleavage [23]. The double-stranded pBR322 plasmid DNA exists in a supercoiled form (form I), which exhibits faster electrophoretic mobility compared to its open circular form (form II, resulting from a single cleavage) and linear form (form III, double-strand cleavage) [24]. As shown in Figure 9, the plasmid DNA consisted of a strong supercoiled form I band and a weak open circular form II band (lane P). Zerumbone had no effect on the DNA mobility compared to the untreated pBR322 plasmid DNA, whereas ZER@MOF-5 exhibited significant retardation of the form I DNA band at all concentrations. Additionally, the conversion of form I to form II was also observed.

### 2.6. HindIII and BamHI Digestion

**Figure 10:**
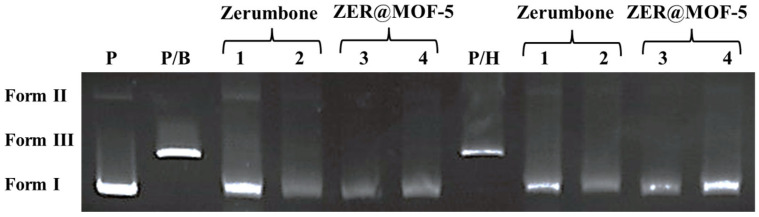
Electrophoretograms of the interaction of pBR322 plasmid with all the compounds, followed by *BamH*I and *Hind*III digestion. (P) untreated pBR322 plasmid DNA; (P/B) pBR322 DNA digested with *BamH*I enzyme; (P/H) pBR322 DNA digested with *Hind*III enzyme. The interaction between pBR322 plasmid DNA and zerumbone (10 mg/mL), followed by *BamH*I and *Hind*III digestion, respectively (line 1). The interaction between pBR322 plasmid DNA and zerumbone (0.625 mg/mL), followed by *BamH*I and *Hind*III digestion, respectively (line 2). The interaction between pBR322 plasmid DNA and ZER@MOF-5 (10 mg/mL), followed by *BamH*I and *Hind*III digestion, respectively (line 3). The interaction between pBR322 plasmid DNA and ZER@MOF-5 (0.625 mg/mL), followed by *BamH*I and *Hind*III digestion, respectively (line 4). The roman numerals I, II, and III indicate form I (supercoiled), form II (open circular), and form III (linear) plasmids, respectively.

The compound/pBR322 plasmid mixture was restricted with *HindIII* and *BamHI* to identify the cleavage site. *Hind*III and *BamH*I are known by recognition sequences A/AGCTT and G/GATCC, respectively [25,26]. When the untreated pBR322 plasmid DNA was digested with *Hind*III and *BamH*I enzymes, only form III (linear) bands were observed, whereas the untreated and undigested pBR322 plasmid DNA gave forms I (supercoiled form) and II (open circular form) bands. The inhibition of *BamH*I and *Hind*III enzyme digestion indicates that the compounds bind covalently to nucleobases in DNA [24]. The *BamH*I and *Hind*III digestion results indicate that the 10 mg/mL and 0.625 mg/mL concentrations of the zerumbone and ZER@MOF-5 bind with A/A and G/G nucleotides (Figure 10).

### 2.7. Free Radical Scavenging Activity

**Figure 11:**
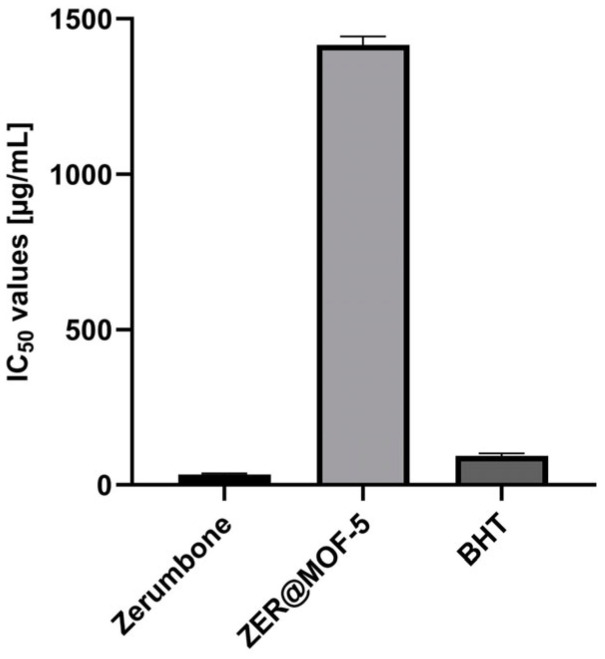
DPPH scavenging activity of zerumbone and ZER@MOF-5. Butylated hydroxytoluene (BHT) was used as a positive control.

Antioxidants are free radical scavengers, which prevent or reduce cellular damage caused by free radicals because of their ability to interact with and neutralize free radicals [27]. The results of the DPPH assay for zerumbone, ZER@MOF-5, and BHT are shown in Figure 11. The DPPH scavenging ability of the zerumbone (IC_50_: 32.89 ± 3.71 µg/mL) was higher than that of the ZER@MOF-5 (IC_50_: 1415.52 ± 27.33 µg/mL) and synthetic antioxidant BHT (IC_50_: 93.29 ± 7.67 µg/mL).

### 2.8. Anticancer Activity

**Figure 12:**
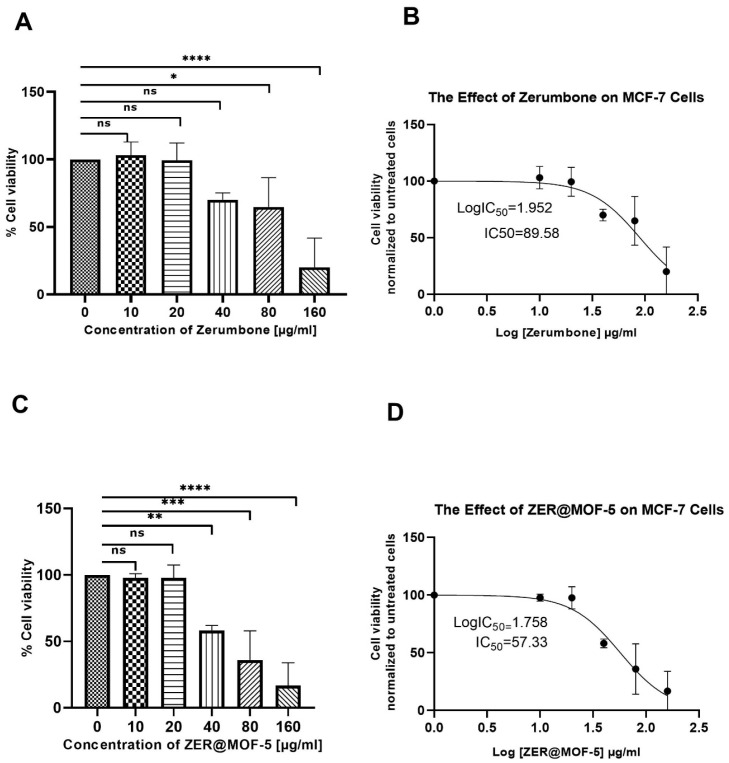
*In vitro* anticancer activities of free zerumbone and ZER@MOF-5 compounds on MCF-7 cells at 24 h post-stimulation. (**A**) The effect of zerumbone on cell viability and (**B**) dose–response curve of cell viability with respect to treatment with different concentrations of zerumbone. (**C**) The effect of ZER@MOF-5 on cell viability and (**D**) dose–response curve of cell viability with respect to treatment with different concentrations of ZER@MOF-5. The results are expressed as cell viability percentage (%) normalized to untreated cells. The data represent the mean values ± SD of three independent experiments performed in triplicate. A one-way ANOVA analysis was applied to determine the statistical differences, followed by Dunnett’s multiple comparisons post-test (ns *p* > 0.05, * *p* ≤ 0.05, ** *p* ≤ 0.01, *** *p* ≤ 0.001, and **** *p* ≤ 0.0001).

Cell viability was assessed using the CCK-8 assay. While zerumbone did not show a significant effect on MCF-7 cell viability at concentrations up to 40 µg/mL, a significant reduction in cell viability was observed at 80 µg/mL compared to the untreated control (*p* ≤ 0.05, Figure 12A). The IC_50_ value for zerumbone was found to be 89.58 µg/mL (95% CI: 68.11–119.9 µg/mL) (Figure 12B). In the case of MCF-7 cells, ZER@MOF-5 significantly decreased cell viability at 40 µg/mL compared to the untreated control (*p* ≤ 0.01, Figure 12C), with an IC_50_ value of 57.33 µg/mL (95% CI: 45.91–72.42 µg/mL) (Figure 12D).

## 3. Discussion

Zerumbone is a crystalline sesquiterpene natural compound with an 11-membered ring that includes a cross-conjugated ketone, which plays a role in its entire biological activity [28]. Zerumbone has a prominent antiproliferative impact on various cancers, such as cervical [29], breast [30], colon [31], lung [32], liver [33], and pancreas [34]. Zerumbone suppresses the growth of cancer cells through the inhibition of major stages of carcinogenesis via the modulation of various signaling pathways and their downstream target proteins [28]. Previous studies have demonstrated that zerumbone has potent *in vitro* antibacterial activity against many bacterial strains. Moreira da Silva et al. [35] reported that zerumbone has antibacterial activity against *Streptococcus mutans*. Another study by Kim et al. [36] proved that zerumbone had antimicrobial and anti-biofilm effects against *Bacteroides fragilis*. Despite the large number of studies in preclinical models showing promising antimicrobial and anticancer properties of zerumbone, its clinical applications are limited by low oral bioavailability, which is associated with low permeability and poor aqueous solubility [4,5]. Therefore, it is of profound significance to develop powerful drug delivery systems for zerumbone to overcome its biopharmaceutical challenges.

To our knowledge, there are no studies investigating the anticancer, antibacterial, antifungal, and free radical scavenging potentials of ZER@MOF-5. The purpose of the present study is to fabricate ZER@MOF-5 and evaluate its anticancer, antibacterial, antifungal, DNA-binding, and free radical scavenging activities. For this purpose, we successfully fabricated MOF-5 and loaded it with zerumbone. The synthesized nanoparticles were characterized using XRD, UV-Vis spectroscopy, FTIR spectroscopy, EDX spectroscopy, and SEM.

Cytotoxicity assay revealed a notable enhancement in the anticancer activity of zerumbone upon its encapsulation into MOF-5. While zerumbone did not exhibit significant cytotoxicity against MCF-7 cells up to 80 µg/mL, ZER@MOF-5 significantly reduced the viability of MCF-7 cells at 40 µg/mL. The IC_50_ value for ZER@MOF-5 was found to be 57.33 µg/mL, which was lower than that of free zerumbone (IC_50_: 89.58 µg/mL). Our findings suggest that MOF-5 plays a crucial role in enhancing the cellular intake or bioavailability of zerumbone, enabling more potent anticancer effects at lower concentrations. Similar results to our study were obtained in studies using MOF-5 as the carrier of different types of anticancer drugs. Chen et al. [16] demonstrated that MOF-5 to load Oridonin exhibited significant cytotoxic activity against the HepG2 cell line, with an IC_50_ value of 22.99 μg/mL. Another study showed that MOF-5 with encapsulated Oleanolic acid enhanced drug release in cancerous cells and strongly inhibited the growth of SK-OV-3 cells [37]. Javanbakht et al. [38] reported that carboxymethylcellulose-coated 5-fluorouracil@MOF-5 exhibited remarkable cytotoxic activity against HeLa cells. Zeolitic Imidazolate Frameworks (ZIFs) are a class of MOFs that are suitable for high-loading drug carriers due to their large surface area. The anticancer activity of CBP@ZIF@DOX@CS-PNIPAAm NP has also been observed by Dashti et al. [39], who proved that the designed NPs exhibit better cytotoxicity against breast cancer cells than free drugs. Also, these NPs were safer than free drugs on healthy cells. Therefore, MOF-5 could be potentially an impressive carrier for oral administration of zerumbone.

The results of our antimicrobial activity experiment showed that the antimicrobial ability of ZER@MOF-5 was limited, whereas free zerumbone exhibited good antimicrobial efficacy. Previous studies have shown that pure MOF-5 exhibits weak antimicrobial activity. For instance, Hu et al. [21] fabricated MOF-5 and Ag@MOF-5 to investigate their antibacterial effects. They reported that the antibacterial activity of MOF-5 was limited compared to Ag@MOF-5. Similarly, Lu et al. [40] demonstrated that the Ag-based MOFs mainly destroy the bacterial membrane, resulting in their death. Silver particles enhance the antibacterial effect of MOF-5, but it is toxic to human cells, limiting their clinical applications. The use of more biocompatible metals, such as zinc, to produce MOFs could be a useful approach to prevent metal ion-based toxicity [41]. Therefore, we employed the encapsulation approach to load zerumbone in Zn-MOF-5, aiming to reduce potential toxicity. However, ZER@MOF-5 exhibited reduced immediate bioavailability of zerumbone due to slow release kinetics, which could explain its lower antimicrobial activity compared to free zerumbone. On the other hand, this sustained release profile could provide significant advantages for prolonged therapeutic efficacy.

Excess free radicals in the human body may lead to damage at cellular and molecular levels and even cause cell death and various diseases, including aging, cancer, diabetes, and cardiovascular diseases [42]. Antioxidants play a pivotal role in the body’s defense against free radicals. Despite well-established therapeutic potentials of antioxidants, their long-term retention poses a challenge for clinical applications [43]. This challenge highlights the need for DDS that can improve the bioavailability of antioxidants. The results of the DPPH assay demonstrated that the IC_50_ of free zerumbone was found to be 32.89 ± 3.71 µg/mL, which was far lower than that of ZER@MOF-5 (IC_50_: 1415.52 ± 27.33 µg/mL). These observations were consistent with a previously reported study, where the antioxidant activity of DOX@Zr-MOF (IC_50_: 1186.14 ± 16.8 µg/mL) was found to be lower than that of free doxorubicin (DOX) (IC_50_: 872.95 ± 11.7 µg/mL) [44]. MOFs encapsulate compounds within their porous crystalline structure, which leads to prolonged circulation time compared to single ligands [43]. Although the unique structure of MOFs may limit antioxidant activity in the short term, it may increase therapeutic efficacy in the long term. However, to fully understand the long-term antioxidant effect of the ZER@MOF-5, further comprehensive *in vitro* and *in vivo* experiments are necessary.

Numerous preclinical studies have suggested that DNA is the main target of many anticancer drugs, which can interact with DNA through both covalent and/or non-covalent interactions [23]. The organic linkers with a conjugated π–electron system facilitate MOFs to interact with small molecules [45]. Recently, research has reported the potential anticancer properties of Zinc(II) compounds. The *in vitro* study performed by Liang et al. [46] demonstrated that Zinc(II) compounds strongly inhibited the growth of various cancer cell lines and interacted with DNA molecules, possibly leading to DNA damage. Zinc(II) compounds interact with DNA molecules through a variety of mechanisms, including groove binding, intercalation, hydrogen bonds, and electrostatic forces [47]. The study by Wu et al. [45] showed that the thymine base interacts with an aromatic organic linker on the MOF UiO-66-NH2 amino functional group via the superposition of π bonds. The results of our DNA-binding experiment show that zerumbone and ZER@MOF-5 have the ability to bind to target DNA. ZER@MOF-5 has also been able to cause a conformational change in DNA.

One of the limitations of our study is that the *in vitro* anticancer activity of ZER@MOF-5 was studied only on MCF-7 cells. Expanding the study to include multiple cancer cell lines is indeed crucial for a comprehensive understanding of the anticancer activity of ZER@MOF-5. Different types of cancer may respond differently to the same drug, so evaluating its efficacy across various cell lines can provide valuable insights into its potential as a broad-spectrum anticancer agent. Another limitation of the study is that the experiments were carried out only using *in vitro* models. While significant progress has been made *in vitro* studies, translating these nanotechnology-based approaches into clinical trials requires *in vivo* studies, such as animal models. These studies can provide a more comprehensive understanding of the safety, efficacy, pharmacokinetics, and potential side effects of ZER@MOF-5 in a more physiologically relevant context. In the next step, a broader range of human cancer cell lines will be used, and *in vivo* animal experiments will be carried out to evaluate the release characteristics, safety, and anticancer activity of ZER@MOF-5, to provide a frame of reference for more effective and targeted therapies.

## 4. Materials and Methods

### 4.1. Chemicals and Reagents

The following reagents and specific materials were applied in the present study: Zerumbone (Sigma Aldrich, Saint Louis, MO, USA), Dimethyl sulfoxide (Sigma Aldrich, Saint Louis, MO, USA), Dulbecco’s Modified Eagle Medium (DMEM) (Gibco, Norristown, PA, USA), 2, 2 Diphenyl 1 picrylhydrazyl (Sigma Aldrich, Saint Louis, MO, USA), Penicillin-streptomycin (Sigma Aldrich, Saint Louis, MO, USA), Fetal bovine serum (FBS) (HyClone, Logan, UT, USA), Dulbecco’s phosphate buffered saline (PBS) (Serox, Mannheim, Germany), Trypsin-EDTA (Capricorn, Düsseldorf, Germany), Cell Counting Kit 8 (WST-8/CCK8) (Elabscience, Houston, TX, USA), Zinc acetate dihydrate (Zn(OAc)_2_·2H_2_O) (Sigma Aldrich, Saint Louis, MO, USA), Triethylamine (Sigma Aldrich, Saint Louis, MO, USA), Terephthalic acid (C_6_H_4_(CO_2_H)_2_) (Sigma Aldrich, Saint Louis, MO, USA), and N,N-Dimethylformamide (DMF) (Sigma Aldrich, Saint Louis, MO, USA). All reagents used were of analytical purity.

### 4.2. Preparation of MOF-5

**Scheme 1:**
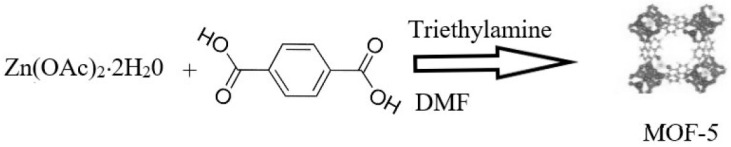
Synthesis of the MOF-5.

MOF-5 was prepared according to previous reports [48]. The synthesis of MOF-5 is described in Scheme 1. In the first step, triethylamine (8.5 mL) and terephthalic acid (5.065 g, 30.5 mmol) were dissolved in 400 mL of DMF. In a separate step, Zn(OAc)_2_·2H_2_0 (16.99 g) was dissolved in 500 mL of DMF. Following that, the zinc salt solution was added to the primary organic solution via stirring for 15 min, which formed a precipitate, and the mixture was stirred for 150 min. After the mentioned time, the precipitate was filtered and immersed in 250 mL of DMF for 24 h. The solution underwent secondary filtration and was immersed in CHCl_3_ (350 mL). The solvent was changed three times over 1 week, after 2 days, 3 days, and 7 days. The solvent was decanted, and it was evacuated to a pressure of 10 mTorr for 24 h. Finally, the product was activated by heating it at 120 °C for 6 h.

### 4.3. Preparation of the ZER@MOF-5

Zerumbone (10 mg) was dispersed in 10 mL of methanol at 25 °C under ultrasonication. Then, the MOF-5 nanoparticles (0.1 g) were immersed in the drug solution and stirred on a magnetic stirrer at 350 rpm at 25 °C for 24 h. The ZER@MOF-5 was centrifuged, washed three times with ethanol, and dried in a fume hood to remove residual ethanol.

### 4.4. Characterization of MOF-5 and ZER@MOF-5

The nanoparticles produced in this investigation were thoroughly analyzed utilizing several analytical methods. XRD analysis of the synthesized MOF-5 powders was performed using a SmartLab Rigaku (Sydney, Australia) diffractometer with Cu-Kα radiation (λ = 1.5406 Å), operated at 40 kV and 30 mA. Diffraction patterns were recorded in the 2θ range of 5–85° to confirm the crystalline structure and phase purity of MOF-5. A spectrophotometer model Agilent/Cary 60 (Santa Clara, CA, USA) was employed to acquire UV–vis absorption spectra. The samples were dissolved in deionized water at a concentration of 0.1 mg/mL. The samples were analyzed for their infrared absorption spectra. The spectra were acquired using KBr pellets and a Varian/660 IR spectrometer (Macquarie Park, Australia), covering the range of 4000–400 cm^−1^. In addition, SEM was used to visually examine the samples. More precisely, a small amount of the sample was placed onto a carbon sheet with holes, and then it was dried to allow for imaging with SEM and mapping of EDX spectroscopy. The images and maps were obtained using a microscope operating at 200 kV, manufactured by SU-1510, Hitachi High-Technologies Corp., Tokyo, Japan.

### 4.5. Determination of Antimicrobial Activity

Zerumbone and the newly synthesized ZER@MOF-5 were tested for their *in vitro* antibacterial properties against different types of Gram-positive bacterial strains, Gram-negative bacterial strains, and antifungal properties against different types of yeast strains (Table 3). Minimum Inhibitory Concentration (MIC) values of the compounds were determined by the microdilution method described in Clinical and Laboratory Standards Institute guidelines [49]. The MIC is the lowest concentration of an antimicrobial agent that inhibits growth as determined visually [50]. Briefly, stock solutions of the zerumbone and ZER@MOF-5 were prepared in DMSO and DMF, respectively. Muller Hinton Agar (100 μL) and Sabouraud dextrose agar medium (100 μL) were added into each well of sterile 96-well microdilution plates for bacteria and yeasts, respectively. Serial dilutions of the compounds were prepared in the medium at 8 different concentrations with a dilution rate of 50%. Approximately 5 µL of bacteria or yeast containing around 5 × 10^5^ colonies were added to each well of the plates, and the plates were incubated for 24 h at 37 °C and 48 h at 30 °C, respectively. Ampicillin, chloramphenicol, and ketoconazole were used as standard antibacterial and antifungal agents. DMSO and DMF were also tested independently for their antimicrobial activity.

The Minimum Bactericidal Concentration (MBC) and the Minimum Fungicidal Concentration (MFC) are the lowest concentration of the compounds that show no growth after a subculture of all the dilutions that showed no growth in the MIC test [51]. The MBC was examined by subculturing 10 μL of content of the microdilution plate on the Mueller–Hinton agar and incubating it for 24 h at 37 °C. The following day, the Petri dish was assessed for the presence of bacterial growth, and the lowest concentration of compounds with no visible growth was recorded as MBC. The MFC was examined by subculturing 10 μL of content from the microdilution plate on the Sabouraud dextrose agar and incubating it for 48 h at 30 °C. The following day, the Petri dish was assessed for the presence of fungal growth, and the lowest concentration of compounds with no visible growth was recorded as MFC.

### 4.6. Determination of the DNA Interaction

The interactions of the zerumbone and newly synthesized ZER@MOF-5 with the DNA were assessed by agarose gel electrophoresis. The supercoiled pBR322 plasmid DNA was incubated with zerumbone and ZER@MOF-5 at decreasing concentrations (10, 5, 2.5, 1.25, and 0.625 mg/mL) for 24 h at 37 °C in the dark. The compound/pBR322 plasmid DNA mixtures were loaded onto the 1% agarose gel; then, the gel was electrophoresed for 2 h at 70 V in 1XTAE buffer. After electrophoresis, the gel was stained in ethidium bromide and then viewed with a UV transilluminator.

### 4.7. Restriction Enzyme Digestion

The supercoiled pBR322 plasmid DNA was incubated with the compounds at concentrations of 0.625 and 10 mg/mL for 24 h at 37 °C and then restricted with 1 unit of the restriction enzymes *Hind*III and *BamH*I for 2 h at 37 °C. After 2 h of incubation, the restricted DNA was electrophoresed in 1% agarose gel at 70 V for 90 min in TAE buffer. The gel was stained with ethidium bromide and then viewed with a UV transilluminator.

### 4.8. Determination of Free Radical Scavenging Activity

Free radical scavenging activities of zerumbone and the newly synthesized ZER@MOF-5 were determined based on the scavenging ability of the stable free radical 2, 2-Diphenyl-1-picrylhydrazyl [52]. The solution of DPPH in methanol 0.04 mg/mL was prepared fresh daily. Stock solutions of zerumbone and ZER@MOF-5 were prepared in DMSO and DMF, respectively. From these stock solutions, working concentrations of 1000, 500, 250, 125, and 62.5 µg/mL were obtained by serial dilution. For each concentration, 100 μL of the sample was mixed with 100 μL of the DPPH reagent in a 96-well microplate. The plate was incubated in the dark for 30 min at room temperature, and then the absorbance was measured at 517 nm. The inhibition percentage of DPPH (I%) was assessed by the following formula:

Scavenging effect (%) = [(Control OD_517_ − Sample OD_517_)/Control OD_517_)] × 100% (1)

The sample concentrations providing 50% inhibition (IC50) were calculated from the graph of inhibition percentage plotted against sample concentrations. Experiments were performed in triplicate. Butylated hydroxytoluene (BHT) was used as a positive control.

### 4.9. Cell Culture

The MCF-7 cell line was obtained from Dr. Canan Eroglu Gunes (Necmettin Erbakan University). Cells were cultured in DMEM with 10% FBS and 1% penicillin (100 U/mL) and streptomycin (0.1 mg/mL) and incubated at 37 °C in a humidified atmosphere of 5% CO_2_. The cells were subcultured into T-flasks two to three times per week and grown to 90% confluency before each assay.

### 4.10. Cytotoxicity Assay

The *in vitro* anticancer activity of zerumbone and the newly synthesized ZER@MOF-5 was studied by the Cell Counting Kit 8 (WST-8/CCK8) (Elabscience, Houston, TX, USA) assay. MCF-7 cells were cultured in 96-well plates in media for 24 h, and on the following day, the MCF-7 cells were treated with increasing concentrations of zerumbone, ZER@MOF-5 (10–160 µg/mL), or media only (control) for 24 h. The plates were incubated for 1 h at 37 °C with 10 µL/well of CCK8 Buffer. A microplate reader was utilized to measure the absorbance of the substrate at OD450 nm. The half maximum inhibitory concentrations (IC_50_) were determined in three independent trials using GraphPad Prism (version 9.4.1). The percentage of cell viability was assessed by the following formula:

Cell Viability (%) = [(Treatment group OD_450_ − Blank well OD_450_)/(Untreated group OD_450_ − Blank well OD_450_)] × 100% (2)

### 4.11. Data Analysis

All data were expressed as the mean ± standard deviation (SD). For the statistical analysis of cell viability, a one-way ANOVA analysis was used to compare untreated and treated groups, followed by Dunnett’s multiple comparisons post hoc test (ns *p* > 0.05, * *p* ≤ 0.05, ** *p* ≤ 0.01, *** *p* ≤ 0.001, and **** *p* ≤ 0.0001).

## 5. Conclusions

Herein, we synthesized MOF-5 and loaded it with zerumbone. Following the characterization process, we then analyzed zerumbone and the newly synthesized ZER@MOF-5 for antioxidants, antimicrobial, DNA binding, and antitumor activities using various *in vitro* studies. Our results demonstrated that the antitumor activity exhibited by ZER@MOF-5 indicates that this compound may be a promising novel candidate for cancer chemotherapy, but further basic and clinical research is required to overcome the current limitations in clinical translation.

## Figures and Tables

**Table 1:** MIC values of the zerumbone and ZER@MOF-5 and reference antibiotic and antifungal agents (MIC values were expressed in µg/mL).

Microorganisms	Compounds		Positive Controls		
	Zerumbone	ZER@MOF-5	Ampicillin	Chloramphenicol	Ketoconazole
*B. cereus* NRRLB-3711	1.25	2500	31.25	125	NS
*B. subtilis* ATCC 6633	>5	>5000	62.5	3.91	NS
*S. aureus* ATCC 25923	1.25	1250	62.5	125	NS
*E. faecalis* ATCC 29212	1.25	1250	31.25	62.5	NS
*E. hirae* ATCC 9790	1.25	2500	62.5	62.5	NS
*S. typhimurium* ATCC 14028	2.5	1250	62.5	125	NS
*P. aeruginosa* ATCC 27853	1.25	1250	>125	>125	NS
*K. pneumaniae* ATCC 13883	2.5	2500	125	15.63	NS
*P. vulgaris* RSKK 96029	1.25	1250	>125	125	NS
*E. coli* ATCC 35218	5	2500	>125	>125	NS
*E. coli* ATCC 25922	0.625	1250	>125	125	NS
*C. albicans* ATCC 10231	1.25	1250	NS	NS	31.25
*C. krusei* ATCC 6258	0.625	1250	NS	NS	<0.98
*C. tropicalis* Y-12968	0.625	1250	NS	NS	31.25

MIC: Minimum inhibitory concentration. NS: Not Studied. Ampicillin and chloramphenicol were not tested against yeast strains, and ketoconazole was not tested against Gram-positive and Gram-negative bacterial strains.

**Table 2:** MBC and MFC values of the zerumbone and ZER@MOF-5 and reference antibiotic and antifungal agents (MBC and MFC values were expressed in µg/mL).

Microorganisms	Compounds		Positive Controls		
	Zerumbone	ZER@MOF-5	Ampicillin	Chloramphenicol	Ketoconazole
*B. cereus* NRRLB-3711	2.5	2500	>125	125	NS
*B. subtilis* ATCC 6633	>5	>5000	62.5	3.91	NS
*S. aureus* ATCC 25923	5	5000	62.5	>125	NS
*E. faecalis* ATCC 29212	>5	5000	125	>125	NS
*E. hirae* ATCC 9790	5	2500	62.5	>125	NS
*S. typhimurium* ATCC 14028	5	2500	63.5	125	NS
*P. aeruginosa* ATCC 27853	2.5	1250	>125	>125	NS
*K. pneumaniae* ATCC 13883	5	2500	125	15.63	NS
*P. vulgaris* RSKK 96029	2.5	1250	>125	>125	NS
*E. coli* ATCC 35218	5	5000	>125	>125	NS
*E. coli* ATCC 25922	2.5	2500	>125	>125	NS
*C. albicans* ATCC 10231	1.25	2500	NS	NS	62.5
*C. krusei* ATCC 6258	2.5	1250	NS	NS	15.63
*C. tropicalis* Y-12968	0.625	2500	NS	NS	125

MBC: The Minimum Bactericidal Concentration, MFC: The Minimum Fungicidal Concentration. NS: Not Studied. Ampicillin and chloramphenicol were not tested against yeast strains, and ketoconazole was not tested against Gram-positive and Gram-negative bacterial strains.

**Table 3:** The bacterial and yeast strains used in the study.

**Gram-positive bacterial strains**	*Staphylococcus aureus* (ATCC 25923) *Bacillus subtilis* (ATCC 6633) *Bacillus cereus* (NRRLB-3711) *Enterococcus faecalis* (ATCC 29212) *Enterococcus hirae* (ATCC 9790)
**Gram-negative bacterial strains**	*Pseudomonas aeruginosa* (ATCC 27853) *Salmonella typhimurium* (ATCC 14028) *Klebsiella pneumoniae* (ATCC 13883) *Proteus vulgaris* (RSKK 96029) *Escherichia coli* (ATCC 35218) *Escherichia coli* (ATCC 25922)
**Yeast strains**	*Candida tropicalis* (Y-12968) *Candida albicans* (ATCC 10231) *Candida crusei* (ATCC 6258)

## Data Availability

Data are contained within the article.
